# The sensitivity of patient-reported outcome measures in surgical and non-surgical care: a systematic review and meta-epidemiological evaluation of randomised controlled trials

**DOI:** 10.1016/j.eclinm.2026.103776

**Published:** 2026-01-29

**Authors:** Mikko Uimonen, Matias Vaajala, Antti Saarinen, Rasmus Liukkonen, Oskari Pakarinen, Juho Laaksonen, Ville Ponkilainen, Ilari Kuitunen, Valtteri Panula

**Affiliations:** a Heart Hospital, Tampere University Hospital, Wellbeing Services County of Pirkanmaa, Tampere, Finland; b Faculty of Medicine and Health Technology, Tampere University, Tampere, Finland; c Department of Orthopaedics and Traumatology, Turku University Hospital, University of Turku, Turku, Finland; d Department of Surgery, Päijät-Häme Central Hospital, Lahti, Finland; e Center for Musculoskeletal Diseases, Tampere University Hospital and Tampere University, Tampere, Finland; f Institute of Clinical Medicine, University of Eastern Finland, Kuopio, Finland; g Department of Pediatrics, Kuopio University Hospital, Kuopio, Finland; h Department of Surgery, Hospital Nova, Jyväskylä, Finland

**Keywords:** PROM, Ceiling effect, Meta-epidemiology, Treatment effect detection

## Abstract

**Background:**

Accumulation of score distribution towards the high end of the measurement scale is an important source of bias related patient-reported outcome measures (PROM). The aim was to evaluate how PROM score distributions, scale boundaries, and sampling variability influence the likelihood of detecting a minimal clinically important difference (MCID) of 10 points between surgical and non-surgical groups in randomised controlled trials (RCTs) of musculoskeletal disorders.

**Methods:**

We did a systematic review and meta-epidemiological analysis of 129 RCT studies comparing surgical and non-surgical interventions in patients with musculoskeletal complaints using a PROM as an outcome measure (1771 group-level PROM measurements) from PubMed and Scopus published until February 26, 2025. Simulations assessed each comparison's likelihood of detecting a difference of 10 points or more.

**Findings:**

The mean difference between groups was 4.6 (SD 7.1) points favouring surgery, with surgical arms scoring higher in 72% of comparisons. The mean likelihood of detecting at least a 10-point difference was 19%, meaning fewer than one in five of such comparisons would detect a true difference. Detection likelihood peaked (35%) at a mean score of 70, declining toward scale extremes. Comparisons with significant observed differences (>10 points, p < 0.05) had a 54% likelihood versus 17% in non-significant comparisons, strongly linking detection likelihood to observed differences.

**Interpretation:**

The majority of the PROM-based RCTs were unlikely to detect differences due to ceiling effects with a constant underestimation of surgical benefit. PROMs with adequate content coverage, better discrimination, and reduced ceiling susceptibility should be selected for clinical practice. Future research should align outcome selection and follow-up timing with expected treatment effects and ensure that measurement properties do not mask meaningful clinical differences.

**Funding:**

None.

**Research in context**

**Evidence before this study**

Before undertaking this study, we considered the existing methodological and empirical literature on the use of patient-reported outcome measures (PROMs) in musculoskeletal randomised controlled trials. Despite recognition of ceiling effects and other PROM-related limitations, we did not identify any previous meta-epidemiological studies that formally quantified how PROM score distributions, scale boundaries, and sampling variability jointly affect the probability of detecting clinically meaningful between-group differences in musculoskeletal RCTs. In particular, no previous work has translated these well-described measurement issues into an explicit, data-driven framework linking observed PROM distributions to the likelihood of detecting a prespecified treatment effect. This gap in the literature motivated the present study.

**Added value of this study**

This meta-epidemiological analysis of 129 RCTs and 1771 group-level PROM measurements provides the first empirical quantification of how PROM score distributions, scale limits, and sampling variability jointly determine the detectability of clinically meaningful group differences. By simulating each comparison's probability of detecting a theoretical 10-point difference, which can be considered as analogous to a power calculation grounded in real data, we demonstrate that the a priori likelihood of detecting a 10-point treatment effect is only 18.5%, decreasing below 20% once mean PROM scores exceed 80. These findings reveal that many apparent “no difference” results in musculoskeletal RCTs may stem not from absent effects, but from measurement limitations inherent to the PROMs themselves.

**Implications of all the available evidence**

When combined with prior research, our findings suggest that the widespread use of PROMs with limited scale range, ceiling compression, and suboptimal content validity poses a substantial risk of underestimating treatment benefits in musculoskeletal RCTs. The low detectability of clinically important differences implies that negative or null findings must be interpreted cautiously, particularly when endpoints cluster near the scale maximum. For clinical practice and policy, these results highlight the need for selecting PROMs with adequate content coverage, better discrimination, and reduced ceiling susceptibility, or adopting modern approaches such as computer adaptive testing. For future research, trialists should align outcome selection and follow-up timing with expected treatment effects and ensure that the measurement properties of the chosen PROMs do not inherently mask meaningful clinical differences.

## Introduction

Popularisation of patient-reported outcome measures (PROM) in outcome assessment has enabled objective measurement of subjective outcomes also in patients with musculoskeletal complaints.^1^^,^^2^ By evaluating the subjective clinical state of a patient through multiple questions, i.e., items, PROMs aim to translate the complex, multidimensional nature of patient's subjective condition into a simplified numerical value. With a basis on this assumption, PROMs have achieved a fundamental role in outcome measurement of intervention trials and a must in US in the process to have medicine approved (reference to FDA). The current study will focus on PROMs for musculoskeletal conditions.

Numerous trials have concluded no benefit of surgery compared to non-surgical interventions or placebo according to a lack of statistically or clinically significant difference in PROM scores between the treatment groups.^3^, ^4^, ^5^ These trials have led to favouring of non-surgical approaches over surgery in multiple, commonly surgically treated conditions.^6^^,^^7^

Theoretical guidelines and checklists have been published to set standards for PROM development and validation processes.^8^^,^^9^ There are numerous PROMs for various conditions and in general they have been described as valid measurement tools. However, if the theoretical guidelines (COSMIN) that have been published since 2010 to set standards for PROM development and validation are followed, many PROMs must be characterised as invalid and inadequate.^8^, ^9^, ^10^ Despite their overwhelming popularity and increasing knowledge of measurement properties, the limitations and potential sources of bias related to PROMs have gained relatively little attention. With regards to the direct consequences of trials concluding no difference between surgical and non-surgical treatment measured using PROMs, the understanding of these limitations appears as unduly weak.

The ceiling effect (i.e., accumulation of score distribution towards the high end of the measurement scale) is an important source of bias related PROMs.^11^ The presence of ceiling effect compromises the sensitivity of a PROM to detect a difference between treatment groups increasing a risk of unjustified “no difference” conclusions. The likelihood of observing a difference between the treatment groups is not solely a property of the exposure treatment, and measurement properties of the PROM that is used, but also related to PROM score distributions at the baseline and the follow up.^11^ Thus, a “no difference” result from a study affected by a ceiling effect should not be interpreted as evidence of true equivalence between treatments, but rather as an indication of an outcome measure lacking sufficient sensitivity to detect a difference.

The objective of this meta-epidemiological study was to quantify how PROM score distributions, scale boundaries, and sampling variability influence the conclusions and specifically the likelihood of observing a theoretical minimal clinically important difference in RCTs comparing surgical and non-surgical interventions in patients with musculoskeletal complaints. We hypothesised that the observed score distributions and the limited range of the PROM measurement scale (i.e., the fact that PROM scores are bounded within a fixed interval such as 0–100, restricting the possible variation in observed scores) favour concluding no difference between the treatment groups regardless of the exposure intervention, and that this in turn leads to a systematic underestimation of the true effect of one intervention over the other. We focused on studies examining surgical treatments in relation to non-surgical treatment.

## Methods

### Search strategy and selection criteria

This meta-epidemiological study has been reported in accordance with the Preferred Reporting Items for Systematic Reviews and Meta-Analyses Meta-Epidemiological extension (PRISMA-ME) guidance. The systematic literature search was conducted in Pubmed (National Library of Medicine) and Scopus (Elsevier) on Feb 26, 2025. The search strategy used for PubMed was as follows: (((“Patient Reported Outcome Measures” [Mesh] OR functionality OR “patient-reported outcomes” OR “subjective outcomes”) AND “Surgical Procedures, Operative” [Mesh]) AND “Orthopaedic Procedures” [Mesh]) AND “Randomised Controlled Trial” [Publication Type], and for Scopus as follows: (TITLE-ABS-KEY (“Patient-Reported Outcome Measures”) OR TITLE-ABS-KEY (functionality) OR TITLE-ABS-KEY (“patient-reported outcomes”) OR TITLE-ABS-KEY (“subjective outcomes”)) AND TITLE-ABS-KEY (randomised AND controlled AND trial) AND TITLE-ABS-KEY (surgery AND operative).

All RCT studies comparing surgical and non-surgical interventions in patients with musculoskeletal complaints using a PROM as an outcome measure were included. Surgical intervention was defined as an invasive intervention using instruments to correct or alter the structural anatomy of tissues, as opposed to conservative treatment, which comprises non-invasive therapeutic approaches, such as physiotherapy or muscle training. Additionally, placebo and sham surgery was considered as non-surgical interventions, as they do not involve a therapeutic surgical modification of anatomy and are designed to simulate the experience of surgery without delivering its active anatomical effects.

Studies were excluded if no extractable numerical PROM data were available, they used only a single-item visual analogue scale as the outcome measure, with no additional PROMs included, or they had non-English full text.

### Ethics

This study was based exclusively on published aggregate data and did not involve direct contact with patients or influence patient care. Consequently, ethical committee approval and informed consent were not required.

### Review process

The results of the literature search were uploaded to Covidence (Veritas Healthcare Inc, Melbourne, Australia) screening software. All authors attended the screening process blinded to each other's decisions. The decisions were based on independent ratings of two authors. In case of disagreement between two screening authors, a third author (MU) made the final decision.

The following information was extracted from the included studies: name of the used PROMs, the score ranges of each PROM (i.e., the minimum and maximum possible scores), sample sizes and mean ages, specific condition and anatomical region of interest, treatment approach (surgery vs. non-surgery), PROM score distribution parameters (means, SDs, medians, IQRs, 95% CIs, standard errors). The PROM scores were collected at all reported time points starting from the baseline to the latest follow-up time-point. If measures of distribution were missing at some time points, the following approach was applied: 1) if distribution data were available at any other time point (six studies), the reported dispersion was extrapolated to the time points where it was missing or 2) if no distribution data were available at any time point (three studies), the average distribution parameters calculated across all samples for the corresponding PROM was used instead.

### Statistics

PROM scores were rescaled to a 0–100 scale, with higher scores reflecting better outcomes. When only a 95% CI or standard error was available, the SD was derived accordingly. If only the median and IQR were reported, the mean and SD were estimated using a simple conversion: the median was treated as the mean, and SD was approximated from the IQR using the formula:

$$SD=\frac{\left(Upper\;bound\;of\;IQR\;-\;Lower\;bound\;of\;IQR\right)}{1.35\;}$$

This approach was based on properties of the normal distribution, where the IQR is approximately 1.35 times the SD. Since the IQR covers the central 50% of the distribution and maintains a consistent relationship with SD in a normal distribution, the approximation was considered sufficiently accurate even when normality could not be assumed, as strict distributional assumptions were not the focus of the analysis.

The scaled PROM scores were pooled by treatment group (surgery vs. non-surgery) and follow-up time point. By assuming a continuous linear scale of PROM score, 95% central distribution of the scores was simulated for each comparison (each PROM, each follow-up time point, each study) regardless of PROM scale limits using formula

95%centraldistribution=Meanscore±1.96×SD

To examine the severity of the ceiling effect, defined as the clustering of scores at or near the upper limit of the scale, the proportion of patients that would have obtained score over 100 if there were no boundaries in the PROM scale, denoting the proportion of patients exceeding the scale (i.e., patients whose clinical status was better than the scale allowed them to express), was estimated by assuming normal distribution under the observed distribution parameters. Proportion of patients exceeding the scale over 15% was considered as significant ceiling effect. Methodology and rationale have previously been discussed in more detail.^11^

The differences in PROM scores between the surgery and non-surgery groups were evaluated by examining their score distributions, specifically, the position of each group on the scale and the mean difference between them. These comparisons were conducted both overall and within strata defined by the overall mean score calculated across all participants. To quantify the chance of observing a theoretical clinically meaningful difference (minimal clinically important difference, MCID) between treatment groups, we performed a Monte Carlo simulation (50 000 draws per comparison) to estimate each comparison's probability of observing a between-group mean difference of at least 10 points given the observed means, variances and sample sizes. For each follow-up time point, let

$${\bar{X}}_{1},s_{1},n_{1}\;\text{and}\;{\bar{X}}_{0},s_{0},n_{0}$$

Denote the sample means, standard deviations and sample sizes in the surgical and non-surgical groups, respectively. Assuming independent normal variation in each arm, the difference of means.

$$D={\bar{X}}_{1}\;-\;{\bar{X}}_{0}$$

was modelled as.

$$D\;∼\;N\left(\mu_{1}-\mu_{0},\frac{s_{1}^{2}}{n_{1}}+\frac{s_{0}^{2}}{n_{0}}\right)$$

Under the null hypothesis of no true treatment effect (μ_1_ = μ_0_), the probability of observing *D* ≥ 10 is

$$P\left(D\geq 10\right)=1\;-\;\Phi \;\left(\frac{10\;-\;\left({\bar{X}}_{1}\;-\;{\bar{X}}_{0}\right)}{\sqrt{\frac{s_{1}^{2}}{n_{1}}\;+\;\frac{s_{0}^{2}}{n_{0}}}}\right)$$

where Φ is the standard normal cumulative distribution function. This likelihood metric aids in planning and interpreting RCTs by indicating under which conditions a prespecified MCID is realistically observable, given the constraints of sampling variability and scale limits. Concretely, a 20% likelihood means that if the same study conditions (sample sizes, variability and true means) were replicated 100 times, only about 20 of those replications would yield an observed group difference of at least 10 points—even when a true difference of 10 points exists. Conversely, in 80% of repetitions the difference would remain below 10 points, “hidden” by sampling variability or ceiling effects of the 0–100 PROM scale. Because ceiling effects constrain scores at the upper limit, and because standard deviations and sample sizes directly determine the width of the sampling distribution, this likelihood estimate sensitively reflects both scale boundaries and study precision. To examine the relationship between the likelihood of detecting a difference and the observed difference, the likelihood distribution was assessed against the per-comparison differences.

Analyses were performed using R version 4.4.2.

### Role of the funding source

This study did not receive any external funding.

## Results

**Fig. 1:**
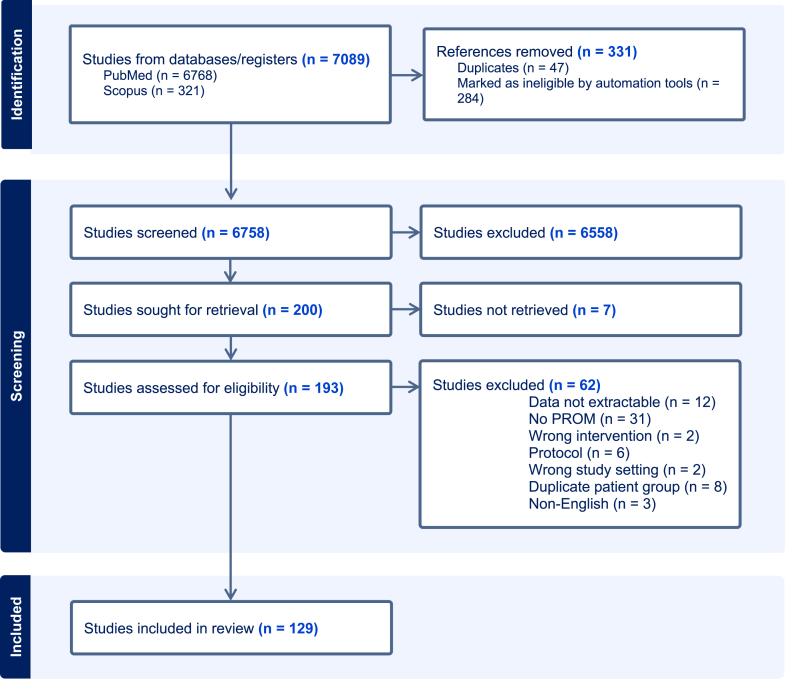
**PRISMA flow chart of the screening process**.

The search yielded 7042 articles, of which, after removing duplicates, 6758 were entered to title and abstract screening after which 200 full texts were screened. Finally, 129 studies were included in the analysis (Fig. 1). Of these, 51 (40%) included elective patients and 78 (60%) included acute patients. The largest proportion of the studies included patients with upper extremity complaints (n = 60, 47%) whereas lower extremity patients were studied in 44 (34%) and patients with back complaints in 25 (19%).

**Fig. 2:**
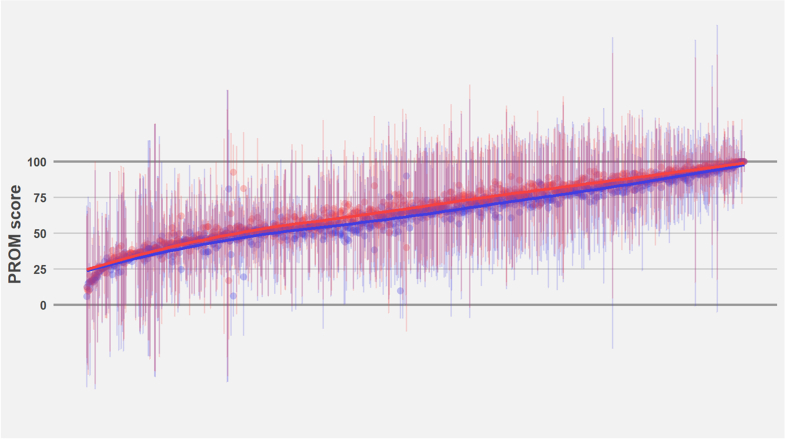
**Distributions of observed PROM scores in surgery (red) and non-surgery (blue) groups across all studies and follow-up time points.** Each point represents the observed group mean score from an individual study, and the whiskers denote the 95% central range of the group-specific distribution assumed for that study. The red and blue lines represent the smoothed mean score trends for surgery and non-surgery groups, respectively, calculated across the PROM measurement scale. X-axis = PROM score comparisons arranged by the observed mean score, Y-axis = the observed score.

There were 1771 group-level PROM measurements in the included studies. Of all comparisons, 72% (496) showed higher scores in the surgery group (Fig. 2). Additionally, when focussing solely on the comparisons at the last follow-up per study, 74% (213/289) showed higher scores in the surgery group. The mean difference between the surgery and non-surgery groups was highest at a mean score of 70–80 and decreased gradually towards the ends of the scale. After excluding comparisons at less than 6 months follow-up the mean score difference was 4.6 (SD 7.1) favouring surgery over non-surgical treatment.

The general mean PROM score (i.e., the mean score for all PROM measurements) was 65 (SD 20). The simulated proportion of patients exceeding the scale was greater than 15% in 20% of surgically treated patient groups (173/871) and in 16% of non-surgically treated patient groups (138/842. In total, 57 out of 129 trials (44%) had at least one measurement exceeding this threshold.

**Fig. 3:**
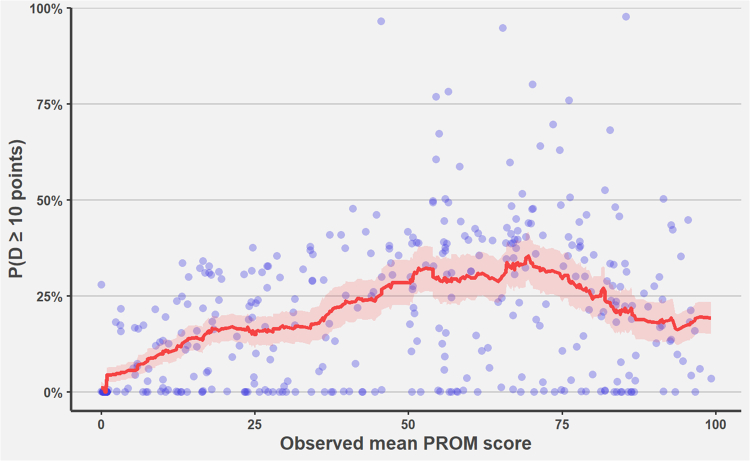
**The likelihood of observing a 10-point difference by the mean surgery and non-surgery groups' PROM score.** The Y-axis shows the likelihood of detecting a 10-point difference per comparison, and the X-axis presents the overall mean score calculated across both groups. Points denote the observed mean scores by comparison and red line shows the rolling mean and 95% CI of the likelihood with a window of 50 comparisons. The likelihood is highest at 50–70 points and decreases steadily towards the maximum score.

**Fig. 4:**
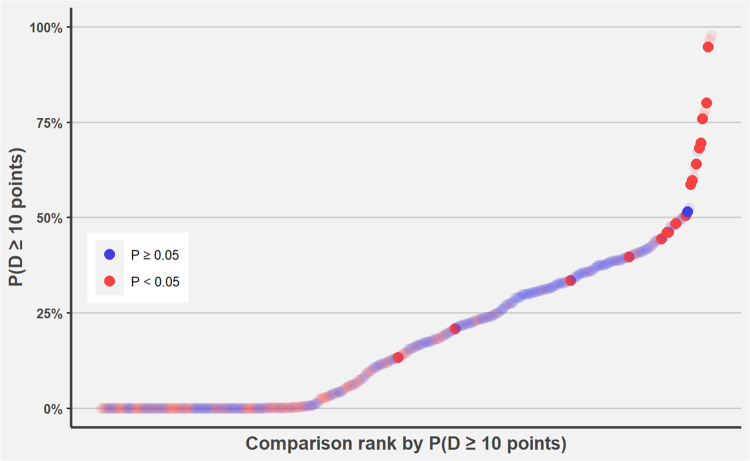
**Distribution of the likelihoods of detecting a 10-point difference across all comparisons arranged by the likelihood.** The Y-axis shows the likelihood of detecting a 10-point difference per comparison, and the X-axis presents all comparisons ordered in ascending likelihood. Red points indicate comparisons for which an independent-samples t-test, using the observed means and SDs, yielded p < 0.05 (statistically significant). Point opacity reflects whether the minimal clinically important difference of 10 points was exceeded between the groups (opaque if exceeded, translucent if not). Note how red, opaque points, representing comparisons with both statistically and clinically significant differences, tend to cluster at higher likelihood values, whereas translucent or non-significant points concentrate toward the lower end of the likelihood scale. This visual pattern demonstrates the link between high detection likelihood and the actual realisation of meaningful group differences. Most comparisons cluster at low likelihood values, suggesting that the majority of studies were unlikely to capture a 10-point difference even when it may have existed.

The mean likelihood of observing a 10-point difference was 19% (SD 19). The likelihood was highest, 35%, at a mean score of 70 and decreased towards the ends of the scale (Fig. 3). From 80 upwards, the likelihood decreased to 20%. This translates to a probability of over 80% that a 10-point difference would not be observed in the study sample, even if it were truly present. Comparisons with observed clinically (>10 point difference) and statistically (p < 0.05) significant difference had a 54% (SD 21) likelihood versus 17% (SD 17) when the difference was clinically or statistically non-significant indicating a strong relationship between the likelihood of observing a difference and the actual observed difference (Fig. 4).

## Discussion

In this large meta-epidemiological analysis of 129 RCTs comparing surgical and non-surgical treatment in musculoskeletal disorders across 1771 group-level PROM measurements, surgical arms demonstrated higher mean scores in almost three out of four comparisons. However, in line with our hypothesis that the observed score distributions and the restricted range of the PROM scale systematically attenuate detectable between-group differences, the a priori probability of detecting our prespecified MCID of 10 points was very low, just 18.5% overall and falling below 20% once mean PROM scores exceeded 80. Such a low theoretical likelihood indicates a severely compromised sensitivity to capture clinically important differences which in turn limits the strength and interpretability of the conclusions drawn from these trials.

As previously shown, the fixed scoring range of PROM instruments places an upper limit on observable outcomes; patients whose true status exceeds what the scale can represent accumulate near the maximum score, which compresses the observable between-group differences, as no further improvement can be measured once the scale ceiling is reached.^11^ Due to this masking effect, a “no difference” conclusion of a trial relying on a PROM with a ceiling effect should be interpreted cautiously. As shown by this study, such conditions are highly prevalent in RCTs comparing surgical and non-surgical treatment in musculoskeletal complaints.

To break the issue down, we simulated each comparison's probability of exceeding the theoretical 10-point MCID given the observed means, variances, and sample sizes. This “likelihood” approach resembles a power calculation calibrated to real data: a 20% chance means that, even when a 10-point true difference exists, only about one in five replications would actually show it. Notably, in comparisons that did achieve ≥10 points with p < 0.05, the likelihood averaged 54% (SD 21), whereas in those with smaller observed differences it was only 17% (SD 17). Further, when the mean score exceeded 80, the likelihood decreased drastically suggesting amplifying constraining effect of scale ceiling. Our findings demonstrate that if the likelihood is less than 45%, observing a difference exceeding MCID would be unexpected. Put another way, a “no difference” conclusion would virtually be inevitable given the low a priori likelihood of detecting an MCID-level gap under these measurement constraints. Under the observed variance, the observed mean difference of 4.6 points corresponds to only an 18.5% likelihood of detecting a clinically meaningful difference. Finally, one should notice that ceiling effects both reduce score variability and limit the observable range, which distorts the estimated probability of detecting meaningful differences. Although the direction of bias varies depending on proximity to the scale maximum, the combined impact of these biases tends to result in an underestimation of the true difference between surgery and non-surgery groups. Thus, our estimates of the likelihood and true group difference may be considered as a bottom line while the real values may be even more pronounced. In summary, the low detection probability often arises from limitations in the outcome measurements and not from the absence of a real effect. This distinction is critical: without recognising the role of ceiling effects, negative or null findings may be misinterpreted as evidence of equivalence, when in reality they may reflect an inability to capture meaningful clinical change.

Our findings underscore that the very choice of PROMs, particularly those prone to ceiling effects, can systematically bias RCT conclusions. However, ceiling compression represents only one of several well-documented limitations of inadequately developed PROMs. Real-world data show that scores often accumulate toward the upper end of the scale following elective orthopedic interventions,^12^ and qualitative investigations have demonstrated that many commonly used PROMs lack sufficient content validity to capture domains that matter to patients.^13^ Moreover, RCTs that employ the most appropriate and well-validated PROMs are significantly more likely to detect between-group differences than those using inadequate instruments (46% vs 22%),^14^ illustrating that poor scale properties directly influence trial outcomes. In our material, these limitations imply that surgical outcomes may be undervalued, leading to potentially unfair comparisons that suggest surgery is no more effective than non-surgical treatment Such misinterpretation carries real-world consequences: patients may endure prolonged disability and extended sick leave, their conditions may progress to chronic stages, and healthcare systems may incur unnecessary costs as less effective treatments are pursued.

The relationship between likelihood and the underlying treatment effect highlights a key point: the goal is to design trials with sufficient sensitivity to detect clinically meaningful differences when they exist. The low observed likelihoods suggest that current PROMs lack the measurement precision to capture these effects. If the outcome measures were more sensitive and less prone to ceiling compression, the true differences between groups, particularly the benefits of surgery, would likely be more readily and more frequently observed. Thus, improving PROM scale properties, rather than accepting small observed differences, is critical for producing reliable and interpretable RCT findings. Going forward, researchers must align outcome selection, scale properties, and follow-up timing with the expected magnitude of surgical benefit, or risk perpetuating underpowered studies that obscure true clinical gains, and ultimately compromise patient welfare. As a targeted remedy to mitigate problems related to ceiling effect, applying computed adaptive testing tools (i.e., dynamic outcome assessment tools that tailor each question based on the respondent's previous answers) instead of traditional PROMs have been proposed.^15^

The present study has several key strengths, alongside limitations that warrant careful consideration. This study includes 129 RCTs and 1771 group-level PROM measurements, representing all available trials using PROMs in musculoskeletal disorders. It applies a unique quantitative framework that integrates real PROM distributions with simulation-based likelihood estimation, providing a previously unavailable method for evaluating the probability of detecting clinically meaningful effects. The study empirically demonstrates how score distributions, ceiling effects, and restricted scale ranges systematically constrain the detectability of between-group differences, thereby highlighting structural limitations that are often overlooked in the interpretation of RCT findings. The work offers actionable insights for clinicians, researchers, and trialists by clarifying when PROM-based RCTs are structurally unlikely to detect clinically meaningful differences and by guiding the interpretation of conclusions that report “no difference. Despite its strengths, this study has several important limitations. First, our approach was theoretical and based on simulations and assumptions on expectable distributions of the patient outcome data. Thus, our findings are not directly convertible to clinical practice. However, we aimed to demonstrate the challenges related to PROMs and the approach was considered appropriate for the task. Second, our meta-epidemiological sample encompasses 129 randomised trials that vary widely in patient populations (elective vs. trauma), follow-up times, sample sizes and, critically, the specific PROM instruments employed. Such clinical and methodological heterogeneity may limit the applicability of pooled estimates of mean scores, variances and simulated detection probabilities across all settings. Third, our likelihood calculations rest on a truncated normal model for the difference of means. Although we truncated simulations at the PROM scale bounds (0–100), real patient-reported scores, especially near the floor or ceiling, may deviate from normality, and any mismatch between the assumed distribution and the true underlying score distribution could bias our estimates. Further, standard p-value thresholds and observed effect sizes capture different dimensions, p-values quantify evidence under a null hypothesis, while our likelihood metric gauges the practicality of detecting a clinically meaningful gap under real-world variability. Fourth, because we relied on Monte Carlo simulation (up to 50,000 draws per comparison) to quantify both ceiling effects and detection likelihoods, residual stochastic variability remains. Even with large simulation counts, chance fluctuation can introduce small errors in individual probability estimates, particularly for comparisons with very small sample sizes. Collectively, these limitations suggest that while our likelihood-based framework offers valuable insights into PROM sensitivity, its numerical results should be interpreted with an understanding of underlying data heterogeneity, modelling assumptions and simulation-related uncertainty. Finally, the protocol of this meta-epidemiological study was not preregistered in any platform. However, since this study was not an interventional study evaluating the effectiveness of any treatment and the aim was to demonstrate a major source of bias in previous studies, preregistration was not considered necessary.

In summary, the majority of the PROM-based RCTs were unlikely to detect differences due to ceiling effects with a constant underestimation of surgical benefit. Conclusions of such trials, particularly those suggesting no difference, should be interpreted with caution.

## Contributors

**Mikko Uimonen:** Conceptualisation, Methodology, Project administration, Supervision, Data curation, Formal analysis, Resources, Software, Visualisation, Writing—original draft. **Matias Vaajala:** Writing—review & editing, Data curation, Validation. **Antti Saarinen:** Writing—review & editing, Validation. **Rasmus Liukkonen:** Writing—review & editing, Data curation. **Oskari Pakarinen:** Writing—review & editing, Data curation. **Juho Laaksonen:** Writing—review & editing, Data curation. **Ville Ponkilainen:** Writing—review & editing, Validation. **Ilari Kuitunen:** Writing—review & editing, Validation. **Valtteri Panula:** Project administration, Data curation, Writing—review & editing, Validation.

All authors read and approved the final version of the manuscript. Mikko Uimonen and Valtteri Panula have verified the underlying data.

## Data sharing statement

The data underlying this study are available from the corresponding author upon reasonable request.

## Declaration of interests

The authors declare that they have no known competing financial interests or personal relationships that could have appeared to influence the work reported in this paper.
